# Editorial: Non invasive BCI for communication

**DOI:** 10.3389/fnhum.2026.1836774

**Published:** 2026-04-27

**Authors:** Sadaf Moaveninejad, Eduardo Santamaría-Vázquez, Jiahua Xu, Camillo Porcaro

**Affiliations:** 1 Biomedical Engineering Research to Advance and Innovate Translational Neuroscience (BRAIN Unit), Department of Neuroscience and Padova Neuroscience Center, University of Padova, Padova, Italy; 2 Centro de Investigación Biomédica en Red de Bioingeniería, Biomateriales y Nanomedicina (CIBER-BBN), Valladolid, Spain; 3 Biomedical Engineering Group, University of Valladolid, Valladolid, Spain; 4 Hvidovre Hospital, Hvidovre, Denmark; 5 Department of Engineering Science, Macau University of Science and Technology, Taipa, China

**Keywords:** artifact handling, asynchronous detection, brain–computer interface, cue-based paradigm, electroencephalography, motor disability, motor rehabilitation, movement-related cortical potential

Brain–computer interfaces (BCIs) has advanced rapidly over the past decades and continues to expand the possibilities of human–computer interaction (Xu et al., 2024). Within this broad landscape, restoring communication for individuals with severe motor disabilities has a particularly strong humanitarian value. Non-invasive BCIs are especially compelling because they do not require surgical implantation. This improves accessibility and safety and supports the potential for wide-scale deployment (Edelman et al., 2024).

Communication-oriented non-invasive BCIs must convert brain signals into reliable communicative outputs, such as selection and spelling, without relying on muscle activity (Kübler, 2020; Martínez-Cagigal et al., 2023). In practice, most systems rely on EEG due to its portability and high temporal resolution, while other modalities are also explored to improve robustness (Xu et al., 2020). However, communication BCIs remain challenging outside controlled settings. EEG has a low signal-to-noise ratio and is sensitive to artifacts and environmental noise (Awais et al., 2024). Performance can also vary substantially across users and sessions, making generalizable decoding difficult (Nijboer et al., 2010). These constraints motivate advances not only in signal processing and machine learning, but also in training and feedback protocols and user-centered design that can support dependable, usable communication in daily life. This Research Topic provides an overview of recent developments in non-invasive BCIs for communication through four articles by 17 authors (Impact: 13k topic views; 8,744 article views; 1,959 article downloads; as of 8 April 2026).

Engaging feedback is often crucial for sustained BCI use, particularly in clinical contexts where motivation and adherence matter. Deuel et al. evaluated a musical auditory feedback BCI, the Encephalophone, in a clinical pilot study. Using EEG-measured motor imagery to enable hands-free musical interaction in real time, the system provides meaningful feedback to support engagement during training. Across three sessions, participants improved pitch-matching accuracy by 15.6 percentage points and increased the number of hits by 58.7%, with accuracy significantly above the chance level (random probability: 19.05%). The authors also report generally favorable self-reported experience (e.g., enjoyment and perceived control). A second Encephalophone-related contribution further explores musical auditory feedback in a clinical setting, reinforcing the broader idea that enjoyable and intuitive interaction can facilitate BCI learning and continued use.

Beyond feedback, communication BCIs ultimately depend on control signals that can support reliable selection in practical interfaces such as spellers. Hernández-Gloria et al. assessed the feasibility of using movement-related cortical potentials (MRCPs) as control signals for a BCI speller in an offline setting. Unlike motor imagery approaches, the study used executed movements: participants selected letters by performing ballistic dorsiflexion of the dominant foot while EEG was recorded from 10 sites centered around Cz. Fifteen healthy participants were recruited (final dataset: 13). The success rate (presence of an MRCP) was approximately 69% in both the control and phrase-spelling conditions, with a slight decrease in the random-letter condition under higher task demands. The authors also highlight that condition-related differences were more evident with Laplacian filtering than with single-site Cz recordings, supporting MRCPs as a candidate signal for speller applications and motivating future validation in real-time settings.

A persistent barrier to translation is that offline performance can overestimate real-world utility when preprocessing choices do not reflect online constraints. Memmott et al. examined artifact filtering with an emphasis on increasing “online parity,” i.e., ensuring that offline preprocessing matches the way data are handled online during closed-loop use. Using P300 rapid-serial visual presentation (RSVP) spelling calibration data from healthy adults (n = 30), they compared conventional filtering applied to the entire dataset with an online-parity approach applied to the segmented epochs available in real time. Across several standard classifiers (including logistic regression and linear discriminant analysis), the online-parity procedure produced small but significant performance improvements, as measured by the Matthews correlation coefficient (e.g., from 0.216 to 0.229 and from 0.334 to 0.343). Overall, the study supports the practical recommendation that training and preprocessing should be performed under conditions that match intended online operation, which may improve the validity of performance estimates and support daily-life communication BCI use.

Reducing calibration time is another practical requirement for movement-based BCIs that aim to operate outside the laboratory, especially in asynchronous settings where users act at times of their choosing and large labeled datasets are often needed. Crell et al. proposed a cue-based paradigm designed to speed up training data collection while preserving key characteristics—and evaluation—of self-paced movement. By emphasizing efficient experimental design, the approach targets the common bottleneck of lengthy data collection. It enables acquisition of 300 cued movement trials in approximately 18 min (ten participants), using a continuously rotating visual cue to reduce the long inter-trial delays typical of conventional cueing. Classifiers trained on these data were evaluated on self-paced movement, achieving an average true positive rate (TPR; percentage of correctly detected movements) of 31.8% at a maximum false-positive rate of 1.0 per minute. Overall, the study illustrates how paradigm design can reduce user burden and accelerate data acquisition for asynchronous movement-intent detection, supporting more feasible real-world deployment.

Overall, this Research Topic highlights that moving communication BCIs toward daily-life use requires advances across the full pipeline: user-centered feedback and training, feasible control signals for communication interfaces, preprocessing choices that reflect closed-loop operation, and data-efficient paradigms that reduce calibration time. Future work will benefit from integrating these directions, for example, pairing online-parity preprocessing with faster data-collection protocols, and evaluating systems in ecologically realistic settings and, where possible, in target clinical populations. Such combined efforts will help bridge the gap between promising demonstrations and robust, user-centered communication BCIs.
